# Feeling well and talking about sex: psycho-social predictors of sexual functioning after cancer

**DOI:** 10.1186/1471-2407-14-228

**Published:** 2014-03-28

**Authors:** Janette Perz, Jane M Ussher, Emilee Gilbert

**Affiliations:** 1 Centre for Health Research, University of Western Sydney, Locked Bag 1797, Penrith South 2751, Australia

**Keywords:** Cancer and sexuality, Psycho-social predictors, Sexual functioning, Quality of life, Communication

## Abstract

**Background:**

Changes to sexual wellbeing are acknowledged to be a long-term negative consequence of cancer and cancer treatment. These changes can have a negative effect on psychological well-being, quality of life and couple relationships. Whilst previous conclusions are based on univariate analysis, multivariate research can facilitate examination of the complex interaction between sexual function and psycho-social variables such as psychological wellbeing, quality of life, and relationship satisfaction and communication in the context of cancer, the aim of the present study.

**Method:**

Six hundred and fifty seven people with cancer (535 women, 122 men) and 148 partners (87 women, 61 men), across a range of sexual and non-sexual cancers, completed a survey consisting of standardized measures of sexual functioning, depression and anxiety, quality of life, relationship satisfaction, dyadic sexual communication, and self-silencing, as well as ratings of the importance of sex to life and relationships.

**Results:**

Men and women participants, reported reductions in sexual functioning after cancer across cancer type, for both people with cancer and partners. Multiple regression analysis examined psycho-social predictors of sexual functioning. Physical quality of life was a predictor for men and women with cancer, and for male partners. Dyadic sexual communication was a predictor for women with cancer, and for men and women partners. Mental quality of life and depression were also predictors for women with cancer, and the lower self-sacrifice subscale of self-silencing a predictor for men with cancer.

**Conclusion:**

These results suggest that information and supportive interventions developed to alleviate sexual difficulties and facilitate sexual renegotiation should be offered to men and women with both sexual and non-sexual cancers, rather than primarily focused on individuals with sexual and reproductive cancers, as is the case currently. It is also important to include partners in supportive interventions. Interventions aimed at improving sexual functioning should include elements aimed at improving physical quality of life and sexual communication, with a focus on psychological wellbeing also being important for women with cancer.

## Background

### Disruptions to sexuality after cancer

It is now widely recognized that cancer and its treatment can have a significant effect on the quality of life of both people with cancer and their family members, in particular their intimate partner [1]. Sexuality and intimacy are important aspects of an individual’s quality of life [2,3], and there is a growing body of evidence to show that cancer can result in dramatic changes to sexuality, sexual functioning, relationships, and sense of self. These changes can be experienced as the most important in the person with cancer’s life [4,5], with the impact lasting for many years after treatment [6,7], often resulting in significant physical and emotional side-effects [8-10].

Sexual difficulties following cancer are primarily the result of the effects of cancer treatments, rather than the disease itself [11,12]. For women, the focus of research has been on the impact of treatments for gynecological or breast cancers, which can result in anatomical changes, such as vaginal shortening or reduced vaginal elasticity [13], pelvic nerve damage, clitoris removal, vaginal stenosis, and fistula formation [14]; and physical changes, such as decreased bodily function [14], fatigue [15], dyspareunia [16], vaginal dryness [17], infertility [18], and post-coital vaginal bleeding [19]. Negative body image or feelings of sexual un-attractiveness [6,20], concern about weight gain or loss [21], loss of femininity [22], as well as alterations to the sexual self [23], can exacerbate the impact of these physical changes. In combination, this can result in changes to women’s response [18], including changes to: desire [18,24], orgasm [21,25], arousal [26], vaginal lubrication [15,17], genital swelling [16] and genital sensitivity [27], leading to decreased frequency of sex [28], and lack of sexual pleasure or satisfaction [29,30].

Research examining men’s sexuality post-cancer has primarily focused on prostate and testicular cancers [31-33]. For example, men with prostate cancer have reported that hormone therapy is like ‘chemical castration’ [34], resulting in erectile dysfunction [35,36], diminished genital size, weight gain, urinary incontinence and bodily feminization [37-39]. Other treatments reportedly result in loss of sexual desire [40], reduced erotic dreams and sexual fantasies [37], decreased orgasmic sensation, and bowel and urinary incontinence [31]. Similarly, following surgery for testicular cancer, men have reported reductions in sexual functioning and enjoyment, fertility concerns, as well as negative body image [41-44]. Rectal cancer has also been associated with reductions in sexual functioning, for both women and men [45,46].

There is some evidence that individuals with colorectal [47-49], colon [50], head and neck [51,52], bladder [53], lymphatic [54,55] and lung [56] cancers can also experience a reduction in sexual interest and sexual activity, changes to body image and feelings of sexual competency, as well as sexual dysfunction, and alterations to sexual self-esteem [50,57,58]. Adult survivors of childhood cancer, across a range of cancer types and treatments, have also been shown to report sexual difficulties and concerns [59,60]. However, previous research on sexual changes after cancer has primarily focused on cancers that directly affect the sexual or reproductive organs, with each study examining a single cancer type, precluding comparison across sexual and non-sexual cancers. The present study will address this imbalance in the research literature through examining changes in sexual functioning and sexual satisfaction, for both women and men, across a range of cancer types, both sexual and non-sexual.

### Pathways to sexual difficulty and distress after cancer

There is a growing body of research examining the association between sexual changes experienced after cancer and quality of life or psychological wellbeing [10,36,47,48,58,61], suggesting that sexual difficulties are associated with lower quality of life, and higher levels of distress. For example, sexual changes have been found to be associated with reduced quality of life or psychological distress in men with prostate cancer [10,36,40,62-64], rare cancers [58], lymphoma [65,66], and colorectal cancer [47]. For women, sexual difficulties have been associated with reduced quality of life or distress in the context of breast cancer [17,67,68], cervical cancer [20,61,69-71], and colorectal cancer [47,48]. Other studies have measured sexual functioning and quality of life as independent outcome variables, but have not examined the relationship between the two [72-75].

A number of factors have been examined as possible predictors of sexual difficulties and psychological distress after cancer, primarily focusing on demographic characteristics such as age [45,47,56,65,76], gender [45,47,48,60], ethnicity [64,77], marital status [68], or education [45,78], as well as the influence of treatment type [45,47,69,73,79]. Older age [45,47,65], and radiation treatment [10,61,69,76] have been consistently associated with lower levels of sexual functioning, with a number of studies also reporting gender differences in demographic predictors of functioning [45,47,79]. However, characteristics of the individual with cancer are not the only predictors of sexual functioning post-diagnosis and treatment. Relationship factors are recognized as having a significant influence on sexual difficulties experienced outside of the cancer context [80], yet the association between relationship factors and sexual adjustment after cancer has been neglected [47]. There is some evidence that quality of the couple relationship is associated with sexual satisfaction and higher levels of sexual functioning [81], and that couples’ quality of life and marital satisfaction are linked [36], in the context of cancer. Successful renegotiation of sexual practices after cancer has also been reported to be associated with couple communication, in qualitative research conducted with cancer carers [82,83]. Nevertheless, these findings are limited, and are primarily based on univariate analysis. The present study will address this limitation in previous research, through conducting multifactorial research to examine the complex interaction between sexual function and psychological wellbeing, quality of life, and relationship satisfaction and communication, in the context of cancer [71,84].

### Relationship communication and context

There is a dearth of previous research examining the influence of couple communication on sexual functioning for people with cancer. There is, however, evidence that the adoption of an open and responsive pattern of couple communication after cancer is associated with lower levels of distress and higher levels of marital satisfaction [85,86], as well as effective emotion and problem focused coping [87], associated with relationship closeness [88,89]. Conversely, many partners are over-protective towards the person with cancer, engaging in “protective buffering” in an attempt to prevent distress [90,91], or “disengaged avoidance” [88], p412, involving complete denial of cancer or its effects. This buffering or avoidance is analogous to the pattern of self-silencing initially identified by Dana Jack [92] as an explanation for women’s greater susceptibility to depression. Self-silencing is characterized as the propensity to engage in compulsive caretaking, pleasing the other, and inhibition of self-expression in relationships, in an attempt to achieve intimacy and meet relational needs [93]. Self-silencing is not a pattern of behaviour unique to women. In a number of studies men have been found to report levels of self-silencing equal to those of women [94,95], or higher than women [96-98]. Differences have also been reported between women and men in patterns of self-silencing [99], and in the relationship between self-silencing and psychological well-being. For example, there is evidence that whilst men report significantly higher self-silencing than women, they also report lower depression [96,100], a finding reported in a recent study of cancer carers [98], whereas self-silencing is positively correlated with depression in women [100]. The present study will examine the association between self-silencing and sexual functioning, as part of a broader multifactorial analysis, in women and men with cancer, as well as partners of a person with cancer, the first study to do so.

Cancer affects not only the person who receives a cancer diagnosis, but also their significant other, leading to the description of cancer as a ‘we-disease’ [88]. Whilst the experiences of partners are often neglected in research on sexuality and intimacy post-cancer [101,102], there is growing acknowledgement of their unmet needs in this area [82,103-105]. Reported disruptions for partners include decreases in sexual drive, fear of initiating sex with their partner, difficulty regaining a level of ‘normality’ within the sexual relationship, sexual communication difficulties, and feeling unwanted and unattractive because of the cessation of sex [34,83,102,106-109]. The present study will, therefore, examine the sexual experiences of partners in comparison with people with cancer, across sexual and non-sexual cancer types, to address this gap in the research literature.

### Study aims and research questions

The aim of this study is to examine the nature of changes in sexual functioning post-cancer and to evaluate the interaction between sexual function and psychological distress, quality of life, and relationship satisfaction and communication. The following research questions are examined. For both men and women with cancer, and their partners, across sexual and non-sexual cancers: How important is sexuality post-cancer? What are the changes in sexual functioning reported before and post-cancer? What psycho-social factors are associated with reductions in sexual functioning post-cancer? What is the relative contribution of psycho-social factors in predicting reductions in sexual functioning?

## Method

### Participants

Six hundred and fifty seven people with cancer (535 women, 122 men) and 148 partners (87 women, 61 men) took part in the study, part of a larger mixed methods study examining the construction and experience of changes to sexuality after cancer. We recruited Australian participants nationally through cancer support groups, media stories in local press, advertisements in cancer and carer specific newsletters, hospital clinics, and local Cancer Council websites and telephone helplines. After reading detailed information describing the research team, the study, consent and complaint procedures, participants completed an online or postal questionnaire examining their experiences of sexuality and intimacy post-cancer. As detailed in the study information sheet, consent to participate was implied through the completion and return of the questionnaire. At the end of the survey, participants indicated whether they would like to be considered to take part in a one hour interview, to discuss changes to sexuality in more depth (additional written consent was obtained for the interviews, with qualitative data reported elsewhere) [110-114]. Two individuals, a person with cancer and a partner, nominated by a cancer consumer organization acted as consultants on the project, commenting on the design, method and interpretation of results. We received ethical approval from the University of Western Sydney Human Research Ethics Committee, and from three Health Authorities (Sydney West Area Health Service, South East Sydney Illawarra Health Service, and St Vincent’s Hospital, Sydney), from which participants were drawn.

### Measures

#### Changes in Sexual Functioning Questionnaire (CSFQ-14)

A 14 item validated instrument that provides a global measure of sexual functioning, using a 5 point Likert scale [115]. It has five subscales identifying different aspects of sexual functioning: desire/frequency; desire/interest; arousal excitement; orgasm/completion; and pleasure, with higher scores indicating higher levels of reported functioning. In reliability testing, the Cronbach alpha coefficients for the total CSFQ-14 score of .90 for the female version and .89 for the male version have been found [115].

#### Hospital Anxiety and Depression Scale (HADS)

A 14 item validated measure developed to measure anxiety and depression in non-psychiatric populations [116]. Each subscale HADSA (anxiety) and HADSD (depression) has a maximum possible score of 21, with a score of between 8 and above recommended for “caseness”, the cut-off for clinical diagnosis. A score of 8-10 is categorized borderline, and a score of 11 and above categorized as abnormal in relation to caseness [117]. In a review of the psychometric literature on HADS, Cronbach alpha coefficients for HADS-A varied from .68 to .93 (mean .83) and for HADS-D from .67 to .90 (mean .82) [117].

#### Medical outcomes study health survey short form (SF-12)

Used to measure health-related quality of life. This measure has been used to evaluate functional states in depressed, chronically ill and healthy populations. The SF-12 is comprised of 12 items, measuring two components: mental health and physical health [118]. Participants rate the degree to which their quality of life is compromised due to their health, on a series of Likert scales. High scores indicate a better quality of life. The SF-12 has good internal consistency and test-retest reliability. Sufficient evidence for the internal consistency of the revised SF-12 as been found (Cronbach alpha coefficients of 0.72 to 0.89) [119].

#### Brief Dyadic Adjustment Scale (DAS)

A 7 item validated instrument which examines relationship satisfaction and cohesion, using a 6-point Likert scale [120]. Higher scores are indicative of higher levels of relationship satisfaction. DAS has shown good internal consistency with Cronbach alpha coefficient of .85 reported [121].

#### Dyadic sexual communication scale

A 13 item scale assessing perceptions of the communication process encompassing sexual relationships, using a 6-point Likert scale, with higher scores associated with better quality of perceived communication [122]. The internal consistency of the DSC has been tested among a cohabitating sample, with a Cronbach alpha coefficient of .83 found [122].

#### The Silencing the Self Scale (STSS)

A standardized questionnaire consisting of 31 items measuring the extent to which individuals endorse self-silencing thoughts and actions in intimate relationships, using a 5 point Likert scale [92]. In addition to a Global score, the four subscales are: Care as Self-Sacrifice (e.g. Caring means putting the other person’s needs in front of my own), Silencing the Self (e.g. I don’t speak my feelings in an intimate relationship when I know they will cause disagreement), Externalized Self Perception (e.g. I tend to judge myself by how I think other people see me) and The Divided Self (e.g. Often I look happy enough on the outside, but inwardly I feel angry and rebellious). High scores indicate greater self-silencing. The internal consistency of total STSS and subscales has been found to range from Cronbach alpha coefficients of .65 to .94 [95].

#### Ratings of sexual importance and activity

Were obtained by participants responding to separate items on the importance of sex as a part of their relationship and as a part of their life on a three point scale: not important, somewhat important, very important. Participants also reported with a yes/no response whether their sexual activities had changed since the onset of cancer.

### Statistical analysis

Univariate analyses were conducted to compare women and men on each of the socio-demographic variables of interest separately for people with cancer (PWC) and partners of people with cancer (PPWC). For continuous variables, one-way ANOVA were conducted with gender as the grouping variable, and the chi square test for independence used for frequency data. Participants reported a range of cancer types, which were categorized into sexual (breast, gynecological, prostate, genito-urinary) and non-sexual (hematological/blood, digestive/gastrointestinal, neurologic, skin and other) for the purpose of analysis. The chi square test for independence was used to test for group differences between sexual and non-sexual cancer types, and women and men, on measures of sexual importance and activity, for both PWC and PPWC. To assess change in sexual functioning after cancer, paired sample t-tests were conducted separately for women and men for PWC and PPWC. Preliminary analyses to multiple regression analyses included independent sample t-tests to assess gender differences in mean scores for all potential predictor variables, and Pearson’s correlations to assess associations between the sexual functioning measures and the criterion total sexual functioning and potential predictor variables for women and men across PWC and PPWC. Finally, to evaluate the relationship between the set of potential predictor variables and the criterion, and identify those variables responsible for the variation in the criterion, standard multiple linear regression analyses were conducted for women and men in the PWC and PPWC samples. Exact alpha levels are reported for all statistical tests, with table notations indicating significance at the .05, .01 or greater than .001 levels where relevant. Ninety-five precent confidence intervals (CI) are reported for effect sizes involving principal outcomes.

## Results

### Descriptive data

Tables 1 and 2 present the sample demographics by gender for the PWC and PPWC samples. Years since first diagnosis of cancer, ethnicity profile, relationship status, and current involvement in a sexual relationship did not differ between female and male PWC. However, men were significantly older, 61.1 versus 50.7 years old, had been in their current relationship longer, 25.7 years versus 19.8 years, were more likely to identify as non-heterosexual, 8.9% versus 3.3%, were less likely to report a sexual cancer, 78% versus 89%, and were less likely to be in remission, 59% versus 81% (Table 1). For the PPWC sample, partner age, length of current relationship, relationship status, sexual identity, involvement in a current sexual relationship, ethnicity profile, years since partner’s first diagnosis of cancer, sexual or non-sexual cancer classification, and stage of disease, did not differ by gender (Table 2).

**Table 1:** Sample characteristics by gender for People with Cancer (PWC)

	**Women**		**Men**		**Test for group difference**	**Significance**	**Effect size**
**Variable**	***n***	***M (SD)***	***n***	***M (SD)***	***F***	***p***	***η2***
Patient age	535	50.7 (10.9)	122	61.1 (14.3)	79.01	<0.001	0.108
Years since first diagnosis	533	4.9 (5.3)	122	5.3 (5.4)	0.53	0.468	0.001
Length of current relationship	515	19.8 (13.7)	118	25.7 (16.8)	16.55	<0.001	0.026
	*n*	%	*n*	%	χ^2^	*p*	*φ*
Ethnicity:					0.51	0.774	0.028
Aust/White European	508	95.7	114	94.2			
Asian	14	2.6	4	3.3			
Other	9	1.7	3	2.5			
Cancer type:					519.19	<0.001	0.364
Breast	425	80	-	-			
Gynecologic	45	8.5	-	-			
Prostate	-	-	87	72.5			
Genitourinary (other)	4	0.8	7	5.8			
Hematological/Blood	23	4.3	14	11.7			
Digestive/Gastrointestinal	11	2.1	4	3.3			
Neurologic	6	1.1	4	3.3			
Skin	8	1.5	2	1.7			
Other^a^	9	1.7	2	1.7			
Cancer classification:					10.52	.001	0.127
Sexual cancer type	474	89.3	94	78.3			
Non-sexual cancer type	57	10.7	26	21.7			
Stage of disease:					27.19	<0.001	0.188
No longer detectable/In remission	430	80.8	71	58.7			
Receiving treatment	16	3.0	7	5.8			
Other^b^	86	16.2	43	35.5			
Relationship status:					3.12	0.374	0.032
Partnered – Living together	414	77.4	96	78.7			
Partnered – Not living together	34	6.4	10	8.2			
Not in a relationship	76	14.2	16	13.1			
Other/Not specified	11	2.1	-	-			
Sexual identity:					405.16	<0.001	0.858
Heterosexual	434	96.7	92	91.1			
Non heterosexual	15	3.3	9	8.9			
Current sexual relationship:					0.99	0.319	0.039
Yes	404	76.2	87	71.9			
No	126	23.8	34	18.6			

Note ^a^“Other” includes: Respiratory/Thoracic, Head & Neck, various, each less than 1%; ^b^ “Other” includes: a new different cancer; active monitoring; outcome not specified.

**Table 2:** Sample characteristics by gender for partners of People with Cancer (PPWC)

	**Women**		**Men**		**Test for group difference**	**Significance**	**Effect size**
**Variable**	***n***	***M (SD)***	***n***	***M (SD)***	***F***	***p***	***η2***
Partner age	87	54.1 (13.5)	61	54.8 (11.1)	0.11	0.738	0.001
Years since partner’s first diagnosis	87	2.9 (2.0)	61	2.9 (2.0)	0.01	0.932	0.000
Length of current relationship	87	25.0 (16.2)	61	23.8 (13.2)	0.24	0.629	0.002
	*n*	%	*n*	%	χ^2^	*p*	*φ*
Ethnicity:					0.85	0.653	0.056
Aust/White European	82	97.6	57	95.0			
Asian	1	1.2	2	3.3			
Other	1	1.2	1	1.7			
Cancer type:					60.10	<0.001	0.297
Breast	4	4.6	29	48.3			
Gynecologic	4	4.6	9	15			
Prostate	35	40.2	2	3.3			
Genitourinary (other)	6	6.9	-	-			
Hematological/Blood	15	17.2	10	16.7			
Digestive/Gastrointestinal	11	12.6	6	10.0			
Neurologic	2	2.3	1	1.7			
Skin	2	2.3	1	1.7			
Other^a^	8	9.2	2	3.3			
Cancer classification:					1.60	0.207	0.104
Sexual cancer type	49	56.3	40	66.7			
Non-sexual cancer type	38	43.7	20	33.3			
Stage of disease:					3.11	0.375	0.095
No longer detectable/In remission	52	59.8	41	67.2			
Receiving treatment	2	2.3	3	4.9			
Other^b^	33	37.9	17	27.9			
Relationship status:					0.12	0.896	0.011
Partnered – Living together	79	90.8	55	90.2			
Partnered – Not living together	8	9.2	6	9.8			
Sexual orientation:					36.16	0.525	0.086
Heterosexual	79	90.8	55	90.2			
Non heterosexual	8	9.2	6	9.8			
Current sexual relationship:					0.12	0.729	0.039
Yes	70	81.4	51	83.6			
No	16	18.6	10	16.4			

Note ^a^ “Other” includes: Respiratory/Thoracic, Head & Neck, various, each less than 1.5%; ^b^ “Other” includes: a new different cancer; active monitoring; outcome not specified.

Measures of sexual importance and sexual activity for PWC and PPWC according to gender and sexual or non-sexual cancer classification are presented in Tables 3 and 4. PWC men were more likely to rate the importance of sex to their relationships and as a part of life as very or somewhat important (97.9% and 96.6%, respectively) than women (86.2% and 78.2%, respectively). There was no significant difference in the reporting of changes in sexual activities since the onset of caner for men (84.6%) and women (76.8%), with the majority of both groups reporting a change. For PPWC, men (91.8%) were more likely than women (84.7%) to rate sex as very to somewhat important as a part of life.

**Table 3:** Sexual importance and activity by gender and cancer classification for People with Cancer (PWC)

	**Women**		**Men**		**Test for group difference**			**Sexual cancer**		**Non-sexual cancer**		**Test for group difference**		
**Item/Variable**	***n***	**%**	***n***	**%**	**χ**^**2**^	***p***	***φ***	***n***	**%**	***N***	**%**	**χ**^**2**^	***p***	***φ***
Sex important part of the relationship:					13.17	0.001	0.158					2.715	0.257	0.072
Very important	148	34.3	45	48.4				162	35.7	30	46.2			
Somewhat important	224	51.9	46	49.5				238	52.4	28	43.1			
Not important	60	13.9	2	2.2				54	11.9	7	10.8			
Sex important part of life:					39.59	<0.001	0.248					9.965	0.007	0.125
Very important	125	23.7	57	48.7				146	26.2	35	42.7			
Somewhat important	288	54.5	56	47.9				304	54.6	37	45.1			
Not important	115	21.8	4	3.4				107	19.2	10	12.2			
Change in sexual activities since onset of cancer:					3.417	0.065	0.074					2.951	0.086	0.069
Yes	394	76.8	99	84.6				432	79.1	55	70.5			
No	119	23.2	18	15.4				114	20.9	23	29.5			

**Table 4:** Sexual importance and activity by gender and cancer classification for partners of People with Cancer (PPWC)

	**Women**		**Men**		**Test for group difference**			**Sexual cancer**		**Non-sexual cancer**		**Test for group difference**		
**Item/Variable**	***n***	**%**	***n***	**%**	**χ**^**2**^	***p***	***φ***	***n***	**%**	***n***	**%**	**χ**^**2**^	***p***	***φ***
Sex important part of the relationship:					5.24	0.073	0.202					0.80	0.672	0.079
Very important	24	31.6	27	50.9				33	42.9	18	35.3			
Somewhat important	41	53.9	22	41.5				35	45.5	27	43.5			
Not important	11	14.5	4	7.5				9	11.7	6	11.8			
Sex important part of life:					9.43	0.009	0.254					2.11	0.348	0.121
Very important	20	23.5	29	47.5				31	35.6	18	31.0			
Somewhat important	52	61.2	27	44.3				43	49.4	35	60.3			
Not important	13	15.3	5	8.2				13	14.9	5	8.7			
Change in sexual activities since onset of cancer:					1.37	0.163	0.098					0.68	0.411	0.035
Yes	62	75.6	40	66.7				64	73.6	38	70.4			
No	20	24.4	20	33.3				23	26.4	16	29.6			

Note. The CSFQ Sexual Pleasure Subscale is only available for ‘after cancer’.

The rankings of the importance of sex to the relationship, and the report of changes to sexual activity post-cancer did not differ between the sexual and non-sexual cancer classifications, with the majority in both groups nominating importance and a change in activities. Non-sexual cancer PWC were more likely to rate the importance of sex as a part of life as very or somewhat important than sexual cancer PWC (87.8% and 80.8% respectively). For the PPWC sample, cancer classification groupings did not differ in reports on sexual importance and activity levels, with the majority in both groups indicating the importance of sex to the relationship and life, and a change in activities, post-cancer. As the impact of cancer upon sexual importance and activity is sufficiently similar across sexual/non-sexual cancer classification groups for the PWC and PPWC samples, subsequent analyses did not test for differences between these groups.

### Sexual functioning according to gender

Paired sample t-tests were conducted for both women and men PWC and PPWC on CSFQ subscales and total scores (Tables 5 and 6) comparing changes in sexual functioning before cancer to after cancer. Cross gender statistical comparison on the CSFQ is not possible, as the scales are specific to men and women. For all CSFQ scores across women and men PWC, sexual functioning scores were significantly lower after cancer than before cancer. Cohen’s effect size values ranged from *d* = 1.228 for the change in women’s sexual desire/interest scores through to *d* = 1.924 for the change in men’s sexual orgasm scores, suggesting a high practical significance in the level of pre to post cancer change.

**Table 5:** CSFQ subscales by gender for People with Cancer (PWC)

	**Women**		**Test for group difference**	**Sign.**	**Effect size**	**Men**		**Test for group difference**	**Sign.**	**Effect size**
**Subscale**	***n***	***M(SD)***	***t***	***p***	***d***	***n***	***M(SD)***	***t***	***p***	***d***
Sexual desire/frequency			21.28	<0.001	1.403			8.73	<0.001	1.388
Before cancer	520	6.85 (1.64)				117	7.40 (1.33)			
After cancer	520	4.92 (1.86)				117	5.86 (1.97)			
Sexual desire/interest			16.63	<0.001	1.228			6.75	<0.001	1.285
Before cancer	516	8.82 (2.51)				115	10.74 (2.12)			
After cancer	516	6.77 (2.62)				115	9.17 (2.44)			
Sexual arousal			21.01	<0.001	1.459			16.96	<0.001	1.807
Before cancer	509	11.17 (3.01)				114	13.69 (1.42)			
After cancer	509	7.27 (3.12)				114	9.26 (2.52)			
Sexual orgasm			18.13	<0.001	1.342			16.32	<0.001	1.924
Before cancer	504	12.04 (2.95)				109	12.29 (1.90)			
After cancer	504	8.72 (3.97)				109	6.88 (3.32)			
Total sexual functioning			13.67	<0.001	1.359			10.49	<0.001	1.547
Before cancer	446	42.22 (7.37)				104	46.55 (5.36)			
After cancer	446	34.73(10.45)				104	36.48 (9.30)			

**Table 6:** CSFQ subscales by gender for partners of People with Cancer (PPWC)

	**Women**		**Test for group difference**	**Sign.**	**Effect size**	**Men**		**Test for group difference**	**Sign.**	**Effect size**
**Subscale**	***n***	***M(SD)***	***t***	***p***	***d***	***n***	***M(SD)***	***t***	***p***	***d***
Sexual desire/frequency			9.75	<0.001	1.125			6.38	<0.001	1.327
Before cancer	80	6.96 (1.44)				58	7.28 (1.47)			
After cancer	80	5.24 (1.62)				58	5.98 (1.57)			
Sexual desire/interest			3.93	<0.001	1.031			1.45	0.152	0.711
Before cancer	77	8.26 (2.58)				55	11.13(5.94)			
After cancer	77	7.38 (2.49)				55	10.04 (2.46)			
Sexual arousal			4.62	<0.001	0.939			3.85	<0.001	1.192
Before cancer	79	11.51 (5.18)				56	8.39 (1.63)			
After cancer	79	9.03 (3.05)				56	7.82 (1.82)			
Sexual orgasm			3.73	<0.001	0.946			2.54	0.014	1.029
Before cancer	77	12.45 (5.45)				52	17.54 (6.10)			
After cancer	77	9.60 (5.21)				52	15.48 (2.69)			
Total sexual functioning			3.39	0.001	1.244			1.057	.296	1.046
Before cancer	70	41.01 (7.28)				50	46.74(13.21)			
After cancer	70	37.13(9.61)				50	44.78 (7.14)			

Note. The CSFQ Sexual Pleasure Subscale is only available for ‘after cancer’.

For the PPWC sample, all CSFQ sexual functioning scores for women were significantly lower after cancer as compared to before cancer scores. For men, all post-cancer sexual functioning scores were lower than before cancer scores with the exceptions of sexual desire/interest and total sexual functioning scores, where differences did not reach statistical significance. In all instances where statistical significance was reached, Cohen’s effect size values indicated a moderate to high practical significance in the observed change from pre to post cancer levels.

### Health related QoL, psychological distress and relationship measures according to gender

Univariate analyses were used to compare women and men on health related QoL, psychological distress and relationship variables of interest. With assumptions of normality and homogeneity of variance met in all instances, independent t-tests were conducted with gender as the grouping variable. Table 7 presents the descriptive data and comparisons between gender for all variables for the PWC sample. For mental health related quality of life, anxiety, dyadic adjustment and sexual communication, and three STSS subscales, results indicated a statistically significant difference for women and men, with women experiencing higher levels of anxiety and ‘externalized self perception’ self-silencing, and lower levels of mental health related QOL, relationship satisfaction and sexual communication. For the STSS subscales ‘silencing the self’ and ‘care as self-sacrifice’, men reported significantly higher self-silencing scores than women. For the PPWC sample, results indicated that women reported a higher ‘externalized self-perception’ STSS subscale score than men, with the differences between women and men on all other variables not being statistically significant (Table 8).

**Table 7:** Means (standard deviations) and comparisons between gender for all potential predictor variables for People with Cancer (PWC)

	**Women**	**Men**				
**Variable**	***M (SD)***	***M (SD)***	***t *** **(655)**	***p***	**95% CI**	***η2***
SF12-Physical component summary	46.33 (10.88)	46.88 (10.23)	-0.51	.609	[-2.67, 1.57]	.000
SF12-Mental component summary	45.25 (11.42)	48.37 (10.42)	-2.77	.006	[-5.34, -0.91]	.012
HADS-Anxiety	9.75 (2.41)	8.68 (3.12)	4.19	<.001	[0.57, 1.58]	.026
HADS-Depression	8.12 (2.15)	7.78 (3.05)	1.65	.099	[-0.73, 0.85]	.004
Dyadic Adjustment Scale (DAS)	27.73 (4.09)	28.57 (3.83)	-1.99	.047	[-1.65, -0.01]	.006
Dyadic Sexual Communication (DSCS)	46.06 (11.63)	48.47 (11.04)	-2.03	.043	[-4.74, -0.73]	.007
Silencing the Self Scale (STSS)						
Externalized self perception	17.34 (5.48)	15.69 (5.21)	3.01	.003	[0.57, 2.73]	0.14
Silencing the self	23.55 (7.74)	26.78 (6.77)	-4.16	<.001	[-4.76, 1.71]	.027
Care as self sacrifice	24.78 (5.93)	31.49 (5.58)	-11.16	<.001	[-7.89, -5.53]	.165
Divided self	16.38 (6.45)	16.58 (6.45)	-0.30	.767	[-1.51, 1.11]	.000

**Table 8:** Means (standard deviations) and comparisons between gender for all potential predictor variables for Partners of People with Cancer (PPWC)

	**Women**	**Men**				
**Variable**	***M (SD)***	***M (SD)***	***t *** **(655)**	***p***	**95% CI**	***η2***
SF12-Physical component summary	51.05 (9.12)	51.28 (8.24)	-0.16	.873	[-3.13, 2.66]	.000
SF12-Mental component summary	45.55 (12.01)	48.32 (12.01)	-1.38	.169	[-6.74, -1.19]	.013
HADS-Anxiety	11.10 (2.49)	11.59 (2.93)	-1.10	.275	[-1.39, 0.40]	.008
HADS-Depression	8.80 (2.02)	8.78 (2.35)	0.06	.956	[-0.73, 0.74]	.000
Dyadic Adjustment Scale (DAS)	28.33 (3.91)	29.29 (3.46)	-1.52	.130	[-2.21, 0.29]	.016
Dyadic Sexual Communication (DSCS)	46.43 (13.04)	48.65 (10.20)	-1.10	.275	[-6.22, 1.78]	.008
Silencing the Self Scale (STSS)						
Externalized self perception	17.76 (8.90)	14.97 (5.78)	2.13	.035	[0.20, 5.39]	.031
Silencing the self	26.05 (8.13)	25.93 (9.54)	0.83	.934	[-2.79, 3.03]	.000
Care as self sacrifice	28.71 (8.39)	30.15 (6.10)	-1.12	.267	[-3.99, 1.11]	.009
Divided self	15.70 (6.49)	15.31 (6.61)	0.35	.724	[-1.80, 2.59]	.001

The correlations between all potential predictor variables and sexual functioning measures according to gender are presented in Table 9 for the PWC sample. For women, all health related QoL, psychological distress and relationship measures were significantly associated with total sexual functioning scores and the majority of sexual functioning subscale scores on the CSFQ. Significant positive correlations were observed for the SF12 subscales, DAS and DSCS and sexual functioning scores, whereas HADS and STSS subscales were significantly inversely correlated with sexual functioning scores. For men in the PWC sample, SF12 health related QoL and DAS measures had significant positive correlations with sexual functioning total scores, as compared to the ‘silencing the self’ and ‘care as self-sacrifice’ STSS subscales which were negatively associated with total sexual functioning.

**Table 9:** Correlations among CSFQ subscales scores and potential predictor variables by gender for People with Cancer (PWC)

		**Women**				
**Variable**	**Sexual desire/frequency**	**Sexual desire/interest**	**Sexual arousal**	**Sexual orgasm**	**Sexual pleasure**	**Total sexual functioning**
SF12-Physical component summary	.14**	.15**	.13**	.14**	.17**	.15**
SF12-Mental component summary	.20**	.07	.21**	.21**	.31**	.22**
HADS-Anxiety	-.11*	-.02	-.12**	-.10*	-.18**	-.16**
HADS-Depression	-.10*	-.05	-.14**	-.13**	-.18**	-.20**
Dyadic Adjustment Scale (DAS)	.28**	.04	.21**	.13**	.37**	.22**
Dyadic Sexual Communication (DSCS)	.35**	.18**	.31**	.31**	.46**	.34**
Silencing the Self Scale (STSS)						
Externalized self perception	-.20**	-.23**	-.26**	-.21**	-.27**	-.20**
Silencing the self	-.24**	-.17**	-.15**	-.26**	-.25**	-.28**
Care as self sacrifice	-.10*	-.18**	-.21**	-.21**	-.08	-.18**
Divided self	-.34**	-15**	-.25**	-.28**	-.40**	-3.32**
			**Men**			
SF12-Physical component summary	.42**	.16	.16	.23*	.36**	.33**
SF12-Mental component summary	.16*	.11	.10	.10	.16*	.17*
HADS-Anxiety	.04	.02	-23**	-20*	-.04	-.11
HADS-Depression	.02	.03	-.19*	-.20*	-.06	-.09
Dyadic Adjustment Scale (DAS)	.21*	.09	.07	.12	.30**	.19*
Dyadic Sexual Communication (DSCS)	.17*	-.10	.06	.13	.43**	.11
Silencing the Self Scale (STSS)						
Externalized self perception	-.08	-.19*	-.03	-.06	-.07	-.15
Silencing the self	-.25**	-.01	-.21*	-.24**	-.25**	-.24**
Care as self sacrifice	-.28**	-.04	-.27**	-.25**	-.13	-.27**
Divided self	-.12	-.03	-.09	-.10	-.29**	-.12

Note. *p < .05; **p < .01, one-tailed.

For the PPWC sample, fewer potential predictor variables were significantly correlated with sexual functioning scores (Table 10). For women, the relationship between relationship satisfaction and sexual communication measures - DAS and DSCS - were significantly positively correlated with sexual functioning total and the majority of the subscales, whilst the ‘care as self-sacrifice’ subscale of the STSS was negatively associated with sexual functioning. Significant positive correlations were observed between the physical health summary score of the SF12 and relationship communication (DSCS) and sexual functioning total and subscales scores, with the STSS ‘silencing the self’ subscale inversely related to sexual functioning for men PPWC.

**Table 10:** Correlations among CSFQ subscales scores and potential predictor variables by gender for Partners of People with Cancer (PPWC)

		**Women**				
**Variable**	**Sexual desire/frequency**	**Sexual desire/interest**	**Sexual arousal**	**Sexual orgasm**	**Sexual pleasure**	**Total sexual functioning**
SF12-Physical component summary	.22*	.24*	.28**	-.14	.15	.12
SF12-Mental component summary	.13	.01	.13	.07	.32**	.13
HADS-Anxiety	.14	.17	.15	.01	.20*	.13
HADS-Depression	-.07	-.03	-.08	-.18	-.15	-.18
Dyadic Adjustment Scale (DAS)	.39**	.17	.24*	.11	.51**	.26**
Dyadic Sexual Communication (DSCS)	.49**	.30**	.37**	.04	.53**	.33**
Silencing the Self Scale (STSS)						
Externalized self perception	-.19*	-.14	.08	-.11	-.23*	-.13
Silencing the self	-.17	-.10	-.18	-.09	-.22*	-.16
Care as self sacrifice	.02	.07	-.15	-.26**	.00	-.19*
Divided self	-.23*	-.08	-.14	-.01	-.42**	-.17
			**Men**			
SF12-Physical component summary	.06	.26*	.27*	.37**	.12	.33**
SF12-Mental component summary	.40**	-.28*	-.14	-.04	.45**	.06
HADS-Anxiety	.29*	-.13	-.15	-.03	.33**	-.03
HADS-Depression	-.04	.17	.05	-.02	-.09	.12
Dyadic Adjustment Scale (DAS)	.11	-.10	.01	.11	.31**	.11
Dyadic Sexual Communication (DSCS)	.49**	-.08	.18	.18	.64**	.34**
Silencing the Self Scale (STSS)						
Externalized self perception	-.22*	.40**	.12	-.04	-.26*	.07
Silencing the self	-.25*	-.09	-.09	-.26*	-.25*	-.23*
Care as self sacrifice	-.14	-.21	.00	-.11	-.06	-.08
Divided self	-.31**	.30*	-.06	-.20	-.48**	-.13

Note. *p < .05; **p < .01, one-tailed.

### Prediction of sexual functioning

Standard multiple linear regression analyses were conducted to evaluate how well health related QoL, psychological distress, relationship satisfaction, sexual communication and self-silencing measures predicted women and men’s total sexual functioning scores for the PWC and PPWC samples. Evaluations of assumptions were satisfactory with no outliners with a standardized residual > 3, and no cases found with a Mahalanobis distance score of *p* < .001 for all analyses performed. To maximize the cases-to-IVs-ratio, potential predictors with non-significant zero-order correlations (as identified in Tables 9 and 10) were excluded in the regression analyses [123]. In all analyses, no multicollinearity amongst predictors was detected with all correlation coefficients < .90. Tables 11 and 12 display the unstandardized regression coefficients (*B*) and intercept, the standardized regression coefficients (*β*), the semipartial correlations (*sr*^2^), *R*^2^, adjusted *R*^2^, *R*, and the confidence limits for significant semipartial coefficients for the conducted regression analyses. Semipartial correlation coefficients are a useful measure for interpretation as they indicate how much each variable uniquely contributes to *R*^2^ (total amount of variance in the criterion accounted for by the predictor variables) over and above that which can be accounted for by the other predictor variables.

**Table 11:** Multiple regression analysis predicting csfq total sexual functioning scores from predictor variables by gender for People with Cancer (PWC)

	**Women**		
**Variable**	**B**	**β**	**sr**^**2 **^**(unique)**
SF12-Physical component summary	.16***	.17	.03
SF12-Mental component summary	.11*	.12	.01
HADS-Anxiety	.26	.06	
HADS-Depression	-.59*	-.12	.01
Dyadic Adjustment Scale (DAS)	.23	.09	
Dyadic Sexual Communication (DSCS)	.23***	.25	.04
Silencing the Self Scale (STSS)			
Externalized self perception	.01	.00	
Silencing the self	-.13	-.09	
Care as self sacrifice	-.11	-.06	
Divided self	.01	.01	
(Intercept)	12.20		
*R*^*2*^	.21^a^		
Total Adj. *R*^*2*^	.22		
*R*	.46***		
95% Confidence limits from 0.14 to 0.28			
		**Men**	
SF12-Physical component summary	.24*	.24	.05
SF12-Mental component summary	.09	.09	
Dyadic Adjustment Scale (DAS)	.20	.08	
Silencing the Self Scale (STSS)			
Silencing the self	-.04	-.03	
Care as self sacrifice	-.42*	-.24	.05
(Intercept)	29.98		
*R*^*2*^	.10^b^		
Total Adj. *R*^*2*^	.12		
*R*	.41**		
95% Confidence limits from 0.04 to 0.30			

a. Unique variability = .09; shared variability = 12%.

b. Unique variability = .10; shared variability = 7%.

*p < .05, **p < .01, ***p < .001.

**Table 12:** Multiple regression analysis predicting CSFQ total sexual functioning scores from predictor variables by gender for Partners of People with Cancer (PPWC)

	**Women**		
**Variable**	**B**	**β**	**sr**^**2 **^**(unique)**
Dyadic Adjustment Scale (DAS)	.20	.08	
Dyadic Sexual Communication (DSCS)	.20*	.27	.05
Silencing the Self Scale (STSS)			
Care as self sacrifice	-.15	-.14	
(Intercept)	28.00		
*R*^*2*^	.13^a^		
Total Adj. *R*^*2*^	.09		
*R*	.36*		
95% Confidence limits from 0.14 to 0.28			
		**Men**	
SF12-Physical component summary	.30*	.35	.11
Dyadic Sexual Communication (DSCS)	.25*	.36	.11
Silencing the Self Scale (STSS)			
Silencing the self	.03	.04	
(Intercept)	16.33		
*R*^*2*^	.23^b^		
Total Adj. *R*^*2*^	.18		
*R*	.48**		
95% Confidence limits from 0.04 to 0.42			

a. Unique variability = .05; shared variability = 8%.

b. Unique variability = .22; shared variability = 0.9%.

*p < .05, **p < .01.

For the women PWC sample, the full regression model significantly explained 22% the variance in total sexual functioning scores, *F*(10, 377) = 9.85, *p* < .001, adj *R*^2^ = .22, 95% CI [-2.38, 26.82]. The relative strength of the individual predictors is presented in Table 11. Squared semipartial correlations indicate that four variables contributed uniquely to the prediction of sexual functioning scores, explaining 9% of the variance. The size and direction of the relationships indicated by the observed standardized regression coefficients suggests higher levels of sexual functioning for women PWC is associated with higher physical and mental health related quality of life and dyadic sexual communication, and lower depression. For the men PWC sample, the linear combination of five predictors significantly explained 12% of the variance in total sexual functioning scores, *F*(5, 95) = 3.68, *p* = .004, adj *R*^2^ = .12, 95% CI [8.95, 51.01]. Only two predictors displayed significant semipartial correlations, suggesting that higher levels of sexual functioning in men PWC is uniquely associated with higher physical health related quality of life and lower levels of the ‘care as self-sacrifice’ subscale of the STSS.

Table 12 displays the results of the standard multiple regressions for the women and men PPWC samples. The full three variable regression model significantly explained 9% of the variance in total sexual functioning scores, *F*(3,73) = 3.50, *p* = .02, adj *R*^2^ = .09, 95% CI 10.54, 45.46], in the women PPWC sample. Only the semipartial correlation between dyadic sexual communication and total sexual functioning scores was significant, uniquely explaining 5% of the variance. The size and direction of the standardized regression coefficient suggests that higher levels of sexual functioning for women PPWC is associated with higher levels of dyadic sexual communication. For the men PPWC sample, the full regression model significantly explained 18% of the variance in total sexual functioning scores, *F*(3, 50) = 4.66, *p* = .006, adj *R*^2^ = .18, 95% CI [-4.28, 36.94]. Two predictors displayed significant semipartial correlations, suggesting that higher levels of sexual functioning in men PPWC is uniquely associated with higher physical health related quality of life and dyadic sexual communication.

## Discussion

This study documented changes in sexual functioning post-cancer for both individuals with cancer, and their partners, across sexual and non-sexual cancers, and demonstrated an association between sexual function and psychological distress, quality of life, sexual communication, and aspects of self-silencing, using a multivariate analyses. Our findings extend previous research examining psycho-social factors associated with sexual changes experienced after cancer, reinforcing, in particular, the importance of physical wellbeing and sexual communication in the context of relationships.

In contrast to previous research that focused on cancers that directly affect the sexual and reproductive organs, in the present study we examined a range of cancer types, finding that sexuality was rated as important, with changes to sexual activities and functioning reported, across sexual and non-sexual cancers. Indeed, individuals with a non-sexual cancer were more likely than those with a sexual cancer to rate sex as an important aspect of their lives. This refutes the belief of some health professionals working in cancer care that sexuality is primarily a matter of concern for individuals experiencing cancers that directly affect the sexual and reproductive organs [124]. It also suggests that information and supportive interventions developed to alleviate sexual difficulties and facilitate sexual renegotiation should be offered to individuals with both sexual and non-sexual cancers, rather than primarily focused on individuals with sexual and reproductive cancers, as is the case currently [125].

Changes to sexual functioning and sexual activities were reported by both women and men in the present study, across people with cancer and partners. This stands in contrast to the notion that sexual changes are primarily experienced by men with cancer [79], and are not an issue of concern for women and partners [126]. The majority of women and men who participated in the study reported reductions in sexual functioning, supporting previous research [8,9,29,44,127]. As the average length of time since diagnosis in the present study was five years, this supports the contention that sexual difficulties can be one of the most enduring effects of cancer diagnosis and treatment [4].

The inclusion of partners in the current study addressed the recognition that partners are often neglected in research on sexuality post-cancer [101,103,128]. Women partners reported reductions across all aspects of sexual functioning, including sexual frequency, interest, arousal, and orgasm, whereas men partners reported reductions in sexual frequency, arousal and orgasm. As the majority of participants were in a heterosexual relationship, this suggests that when a man has cancer, all aspects of sexual functioning are diminished for the woman partner. In contrast, for this sample, when a woman has cancer, her male partner may maintain sexual interest, but experience reduced functioning in other aspects of the sexual relationship. The question of whether this means that sexual changes are more problematic for men than for women partners is deserved of further investigation. It has previously been reported that women do not consider changes in sexual desire in their male partner with prostate cancer a major concern, as their primary focus is survival of their husband [126]. However, qualitative reports in the present study suggested that sexual changes were problematic for many women partners, as was also the case for male partners [111,112,129]. This suggests that the sexual concerns of partners, as well as those of people with cancer, should be part of the agenda of health professionals working in cancer care [82,83].

Previous research has suggested that changes in sexual functioning are associated with reduced quality of life and psychological distress [10,36,47,48,58,61], relationship satisfaction [36], and couple communication [82,83]. This was confirmed in the correlational analysis in the present study, where significant associations were found between sexual functioning and a range of psychological and relational variables. There were commonalities and differences across men and women, and across people with cancer and partners, in the strength of the associations between sexual functioning and psycho-social variables. This is reflected in the multivariate analysis, which examined the relative importance of each of the predictor variables.

The finding that the physical component of QoL was a significant unique predictor of sexual functioning for men and women with cancer, as well as male partners, suggests that physical wellbeing is an important determinant of sexual wellbeing after cancer. Deterioration in physical wellbeing may be associated with cancer treatment in the case of men and women with cancer [11,12]. The physical consequences of aging may also be a factor affecting physical QOL and sexual functioning, suggested by previous reports that sexual frequency and functioning declines with age for some individuals [130,131]. However, the majority of participants in the present study described sexuality as an important aspect of their life and relationship, supporting previous reports that many late middle aged and older adults are sexually interested and active [132,133]. This refutes the widely held social construction that older adults are not interested in sex [134], a construction adopted by some health professionals working in cancer care [124,135], and suggests that information and support for sexual difficulties experienced after cancer should be offered to patients and their partners regardless of age.

In addition to physical QoL, for women with cancer, depression and mental QoL were unique predictors of sexual function. The association between psychological distress and sexual functioning is well documented, for both women and men in the general population [136,137], with a bi-directional relationship existing between distress and sexual difficulties, with both potentially sharing a common etiology [138]. The gender difference in psychological predictors of sexual functioning found in the present study may reflect the lower levels of mental QoL reported by women with cancer in comparison to men. However, women did not report significantly higher rates of depression than men, the other unique predictor, and whilst they did report higher rates of anxiety, reflecting gender differences in anxiety in the general population [139], this was not a significant predictor of sexual functioning. It has previously been reported that marital satisfaction is associated with sexual functioning [140] and that women are more likely report marital dissatisfaction than men, which is associated with depression [141]. In the present study, relationship satisfaction was significantly correlated with a greater number of sexual functioning subscales for women than for men, suggesting that relationship satisfaction may be a factor in gender differences in psychological distress and sexual functioning after cancer. Gender differences in the relationship between psychological distress, QoL, sexual functioning and relationship satisfaction in the context of cancer are deserved of further examination.

Previous research has demonstrated an association between couple communication and adjustment in the context of cancer [86,87,91,142]. Couples who are mutually responsive, attend to each other’s needs, and talk openly about their stress, are more able to engage in effective coping [87], which allows them to find benefits in the cancer experience, such as personal growth and relationship closeness [88,89]. This pattern of mutual communication has also been found to be associated with lower levels of distress for patients and partners, and higher levels of marital satisfaction [86,143,144]. In the present study, sexual communication was a significant predictor of sexual functioning for women with cancer, and for men and women partners, with higher levels of communication associated with higher levels of functioning. This supports the findings of a previous study conducted with men who had prostate cancer and their partners [39], and suggests that talking about sexual changes and concerns can play a major part in addressing difficulties. In this vein, in previous qualitative research conducted with partners of a person with cancer, sexual communication was identified as a key factor in sexual re-negotiation, allowing couples to discuss alternative forms of sexual intimacy in the face of physical changes experienced after cancer [82,107]. The importance of sexual communication in overcoming sexual difficulties and developing new sexual practices was also identified in accounts of men and women with cancer, and partners, in the qualitative component of the present study [113]. These findings reinforce the importance of recognizing the inter-subjective nature of sexual difficulties experienced after cancer, and the importance of researchers and clinicians adopting a relational approach [88,145,146], including the development of couple-based information and support for sexual difficulties [147].

In the present study, sexual communication was not a significant predictor of sexual functioning for men with cancer in the multivariate analysis, even though significant associations were found in the correlation analysis between sexual communication, sexual desire and pleasure. In the general population, there is evidence that women are more likely than men to discuss sexual health concerns [148], and that absence of relationship communication on the part of male partners causes greater distress for women [149]. It has also been suggested that women’s wellbeing is more strongly associated with relational factors than men’s [150,151]. However, sexual communication was a predictor of sexual functioning for male *partners* in the present study, suggesting that there is an interaction between gender, the role of patient/partner, sexual functioning and sexual communication. The nature and implications of this interaction is deserved of further investigation.

Relational factors are also implicated in the finding that the ‘care as self-sacrifice’ subscale of self-silencing was a unique predictor of sexual functioning in men with cancer, with lower care as self-sacrifice associated with higher levels of functioning. In contrast to previous research examining self-silencing and psychological distress in cancer carers [98], there was no difference between men and women partners on levels of self-silencing in the present study. However, men with cancer reported significantly higher levels of ‘care as self-sacrifice’ and ‘silencing the self’ than women with cancer, in line with previous reports [96,97,152]. The ‘care as self-sacrifice’ subscale, the unique predictor of sexual functioning in the multivariate analysis, assesses the propensity to put the needs of others before the self. This suggests that men who put their partner’s needs first report lower sexual functioning. Reports from the qualitative aspect of the present study [114], as well as previous research on women with cancer [153] and women partners [82], suggests that some women are engaging in coital sex in order to please their partner, rather than to fulfill their own needs, even when coital sex results in pain or discomfort. Men who are attuned to the woman’s absence of desire or response may be less likely to initiate sex, and therefore to report lowered sexual functioning. However, relational closeness may be maintained through non-coital practices, as evidenced in the qualitative findings resulting from the present study [113], suggesting that lowered sexual functioning as measured by a standardized scale may not be a wholly negative experience. This suggests that researchers and clinicians need to be aware of the meaning of sexual functioning for men and women affected by cancer, and the way in which this meaning is negotiated in the context of relationships.

## Conclusions

The strengths in the present study were the inclusion in the participant sample of sexual and non-sexual cancers, men and women, and people with cancer and partners, across a range of relationship contexts. The utilization of a range of standardized measures to evaluate sexual functioning, psychological distress, and relationship satisfaction and communication is also a strength. A limitation was the cross sectional nature of the study, which did not facilitate examination of changes over time, and the self-selected nature of the sample, potentially excluding individuals for whom sexual changes after cancer were not evident, or were not a concern. A further limitation was the greater proportion of women with breast cancer in the sample, compared to other cancer types. This was the result of the positive response to requests to take part in the research on the part of women with breast cancer, which may reflect the greater willingness of women to talk about sexual difficulties combined with the impact of breast cancer on women’s sexuality [8,15]. However, we also received a good response from individuals and their partners affected by other types of cancer, including those with prostate, hematological, gastrointestinal, neurological, skin, respiratory, and head and neck cancers, substantiating our conclusion that sexual changes are experienced by individuals across a range of cancer types, and that relationship context is an important predictor of sexual functioning. This suggests that clinicians and researchers need to acknowledge the psycho-social context when dealing with sexual changes experienced after cancer, alongside physical wellbeing, across the whole spectrum of cancer care.

Changes in sexual activities and sexual functioning may be one of the most enduring negative effects of cancer treatments [5], but distress associated with such changes can be alleviated, and strategies of sexual re-negotiation developed [113], through the provision of information and professional support. However, there is evidence that discussion of the nature and causes of sexual changes after cancer, as well provision of information about effective coping strategies, is often absent in clinical consultations [124,135], in particular for women and for individuals with a non-sexual cancer [135]. As a result, sexual concerns remain unaddressed [154]. Equally, whilst a range of one-to-one and couple interventions have been developed to address sexual difficulties after cancer [125,155-158], these are primarily focused on the functioning of the body for individuals with breast, prostate or gynaecological cancer. The findings of the present study suggest that there needs to be an expansion of such support into non-sexual cancers. Details of specific strategies that can be adopted in raising sexual issues in a clinical context are now widely available [159-162]; these need to be utilised as part of normal clinical practice, with both patients and their partners, across all types of cancer. One example is the PLISSIT model of providing permission, limited information, specific suggestions, and intensive therapy [163]. This model facilitates various levels of intervention, as deemed appropriate for the patient and their partner: at the most basic level legitimating the discussion of sex and intimacy within couple relationships and with health professionals (“permission”); providing “limited information” through discussion or written information; “specific suggestions” about changes to sexuality following cancer; followed by referral for “intensive therapy” if needed. At the same time, information and checklists provided to people with cancer can facilitate their raising the subject of sexuality with clinicians, which can alleviate concerns that such discussion is unwanted e.g. [164,165].

## Competing interests

The authors declare that they have no competing interests.

## Authors’ contributions

JP, JMU and EG designed, planned and coordinated the study with significant input from The Australian Cancer and Sexuality Study Team (ACSST)^1^. JP performed the analysis and initial interpretation of the results with JU assisting in the interpretation of data. JMU and JP drafted the manuscript with EG revising it critically for important intellectual content. All authors read and approved the final manuscript.

^1^ACSST members include: Gerard Wain (Westmead Hospital), Gill Batt (Cancer Council New South Wales), Kendra Sundquist (Cancer Council New South Wales), Kim Hobbs (Westmead Hospital), Catherine Mason (Nepean Hospital), Laura Kirsten (Nepean Hospital) and Sue Carrick (National Breast Cancer Foundation).

## Pre-publication history

The pre-publication history for this paper can be accessed here:

http://www.biomedcentral.com/1471-2407/14/228/prepub
